# Program of quality improvement for extracorporeal blood purification therapies in the intensive care unit

**DOI:** 10.1007/s10877-025-01396-7

**Published:** 2025-12-12

**Authors:** Matteo Cecchi, Diego Pomarè Montin, Antonio Fioccola, Vittorio Bocciero, Caterina Scirè Calabrisotto, Filomena Autieri, Manuela Benelli, Andrea Geppetti, Zaccaria Ricci, Stefano Romagnoli, Gianluca Villa

**Affiliations:** 1 Department of Health Sciences, Section of Anesthesiology, Intensive Care and Pain Medicine, University of Florence, Florence, Italy; 2 Department of Anesthesia and Intensive Care, ASST Santi Paolo e Carlo, San Paolo University Hospital, Milan, Italy; 3 Unit of Accreditation, Quality and Risk Management, AOU Careggi, Florence, Italy; 4 Department of Civil and Environmental Engineering, University of Florence, Florence, Italy; 5 Department of Anesthesia and Critical Care, Meyer Children’s University Hospital, IRCCS, Florence, Italy; 6 Department of Anesthesia and Intensive Care, Section of Oncological Anesthesia and Intensive Care, AOU Careggi, Florence, Italy

**Keywords:** Risk, SWOT analysis, Education, High-fidelity simulation, Registry

## Abstract

Critically ill patients often require complex extracorporeal treatments, such as extracorporeal blood purification (EBP). At the bedside, there can be reluctance or uncertainty about when to initiate EBP, and there is no standard agreement on which goals to pursue, what prescriptions to use to achieve those goals, or which recommendations to follow to prevent complications. Furthermore, an accurate analysis of why clinical goals are not achieved or how often the patient should be reassessed to readjust the EBP prescription is not currently standardized. This narrative review describes the main actions characterizing a quality improvement program for EBP in the ICU, which took place at the University of Florence and was subsequently adopted at the national level. The pillars of this program were: (1) definition, implementation, and dissemination of information and communication technology tools aimed at objectively measuring results at the bedside, supporting dynamic prescribing and precision medicine, and promoting advances in knowledge in this field; (2) creation of a national multi-professional network of clinical users and researchers in EBP; (3) promotion and maintenance of technical and non-technical skills in EBP based on the reformulation of advanced academic training in this field.

**Supplementary Information:**

The online version contains supplementary material available at 10.1007/s10877-025-01396-7.

## Introduction

Critically ill patients often require complex extracorporeal treatments, such as extracorporeal blood purification (EBP) therapies, to support organ dysfunction [1, 2]. Extracorporeal blood purification (EBP) refers to a set of extracorporeal techniques designed to remove or modulate circulating solutes, toxins, and inflammatory mediators in order to restore physiological homeostasis and support organ function. In critical care, EBP encompasses kidney replacement therapies—including hemofiltration, hemodialysis, and hemodiafiltration—as well as adsorption-based and hybrid modalities applied in acute kidney injury and sepsis-related multiple organ dysfunction [3–5].

Acute kidney injury (AKI) is one of the most frequent organ dysfunctions observed in the intensive care unit (ICU) [6]. Thus, kidney replacement therapy (KRT) is the most commonly used EBP in critical care settings [7]. AKI affected 36.1% of patients in a major Australian ICU study. Although the study lacked specific KRT data for AKI cases, the authors’ reference to a 4% KRT rate in critically ill patients generally still points to a considerable association between AKI and adverse patient outcomes in this setting [8, 9]. Critical care physicians often require specific technical knowledge and a multidisciplinary approach to prescribe and administer EBP properly. The complex pathophysiological disorders of critically ill patients and the tremendous technological advances that have characterized EBP devices and their disposables over the past decade make managing these treatments a highly specialized and challenging endeavor [10, 11]. Therefore, clinical and technical expertise is needed among clinicians adopting EBP. Uncertainties may arise among users of bedside EBP about when to initiate it and what goals to pursue, which prescription to use to achieve those goals and how to prevent complications, why clinical goals may not be achieved, or how often to reassess the patient to adjust the EBP prescription.

Quality improvement and education programs are essential. Patient outcomes should be continuously assessed through local measures. In addition, practices should be promoted to continuously improve clinical outcomes, achieve a deeper awareness of the real-life practice and effectiveness of EBP, efficiently utilize limited resources, and apply social equity. Finally, knowledge and practical skills in clinical management, technical/engineering updates, and economic/logistic management related to the use of EBP are needed to improve patient outcomes.

This article describes the strategic plan adopted by many Italian Centers to implement safety and quality improvement programs focused on EBP performed in the ICU. In particular, this narrative review describes the main actions characterizing the safety and quality improvement program for EBP performed in the ICU, which took place at the University of Florence and was followed by its adoption at the national level.

The pillars of this program were: (1) definition, implementation and diffusion of Information Communication Technology (ICT) tools aimed at objectively measuring outcomes at the bedside, supporting dynamic prescription and precision medicine, promoting advances in knowledge in this field; (2) creation and support of a national multi-professional network of EBP clinical users and researchers; (3) promotion and maintenance of technical and non-technical skills on EBP based on the reformulation of the advanced academic education in this field.

## The ICT tool supporting acute renal replacement therapies (ARRT): the ARRT registry

The ARRT Registry is a research tool and decision support system provided by the University of Florence to assist clinical practice and improve knowledge in the field of EBP. It is a web-based platform that is freely available for physicians caring for critically ill patients requiring EBP. Internet access is possible via tablet, smartphone, or any other personal or hospital internet-connectable device. The ARRT Registry was conceived and developed within the Department of Health Sciences at the University of Florence and officially launched on 28 June 2019 (ClinicalTrials.gov NCT03807414). A multidisciplinary Scientific Board (intensivists, nephrologists, nurses, data scientists) oversees the registry and performs annual and ad-hoc reviews of its scope, data elements, and analytical approaches. In its preliminary version, the ICT tool was developed to objectively record patients’ clinical and environmental characteristics undergoing blood purification and map the Centers involved in EBP practice for research purposes. During this phase, the objectives of the ARRT Registry were to describe the population of critically ill patients treated with EBP and to explore current practice in terms of disposables, prescriptions, and clinical outcomes. Guidelines exist, but specific recommendations are still missing due to the high heterogeneity of patient populations and blood purification approaches. Within these premises, the ARRT Registry could objectively describe the current practice and underline the different approaches and outcomes obtained by different centers and prescriptions for different patients. Inferential statistical analysis of advanced computational biomedical research will identify which treatment settings applied to specific patients’ phenotypes are significantly associated with better clinical outcomes. Advances in knowledge in this field are thus the primary objective of this tool.

The ARRT Registry has recorded local policies and strategies of EBP, along with clinical and environmental outcomes. The ARRT Registry automatically processes recorded data to calculate derived clinical indices and to interact in real time with users, while preserving all original entries unmodified. This functionality increases physicians’ awareness of trends and associations among variables and facilitates outcome evaluation at the bedside. For example, long-term survival can be a significant clinical outcome for younger trauma patients, and the adoption of an expensive, invasive, and highly complex treatment such as an EBP should undoubtedly carefully consider such an outcome. For elderly oncological patients with multiple comorbidities admitted for acute critical illness (e.g., sepsis), long-term survival cannot be realistically considered as a significant outcome. Instead, organ function recovery and patient autonomy preservation at ICU discharge (e.g., dialysis independence) might be recognized as a reasonable patient-centered clinical outcome. Similarly, reductions in the time of vasopressor or mechanical ventilation requirements, ICU length of stay, or bacterial superinfection may be significant environmental outcomes that should be considered, and their association with EBP management should be carefully assessed. The ARRT database may enable physicians to consider these outcomes and encourage them to regularly revise local policies and practices to assess tactics affecting modifiable elements.

**Fig. 1:**
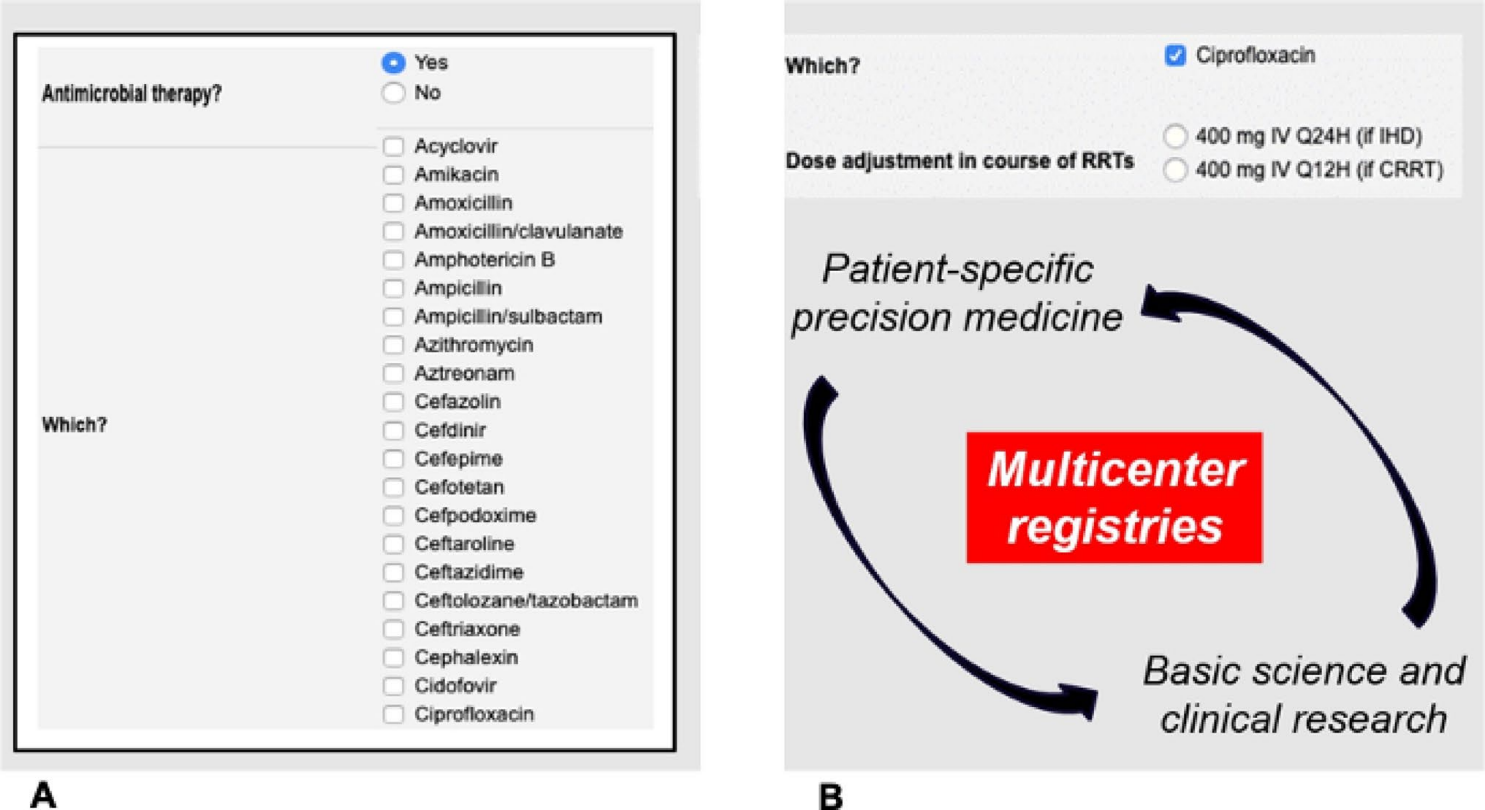
Web-based platform for clinical decision-making. (**A**) Clinical data-entry interface. Example of the web-based platform used to assist physicians in prescribing and administering extracorporeal therapies and related treatments according to current guidelines and best practices. When antimicrobial therapy is selected, the system displays a list of available drugs and recommended dosages automatically adjusted for renal-replacement modality. (**B**) Conceptual framework linking bedside data to research. Schematic representation of how the ARRT digital infrastructure connects patient-specific data collection with multicentre research and quality-improvement activities, bridging precision medicine and translational science

During the last five years, the ARRT Registry has undergone multiple connectivity and computational features improvements, making this web-based platform a valuable tool for clinical decision-making (Fig. 1A and B).

Electronic sniffers, decision support systems, or other ICT tools have been introduced in clinical medicine to support physicians at the bedside, particularly in promoting timely diagnosis and precision medicine [12]. The inclusion of calculators into the ARRT Registry has allowed this web-based tool to implement strategies to support the physician in prescribing and delivering the most adequate EBP treatment for each specific patient per current guidelines and best practices. In particular, advanced calculators continuously and automatically manipulate patient data recorded within the ARRT Registry to support diagnosis and staging of clinical syndromes (e.g., Berlin and KDIGO Criteria, respectively for acute respiratory distress syndrome and AKI), prognostic scoring systems (e.g., APACHE II or SOFA score) or treatment settings (e.g., ventilator or pharmacological therapies readjusted for multiple anthropometric/physiologic/pathologic patient’s parameters and environmental factors). The ARRT decision-support rules are derived from international guidance (e.g., KDIGO AKI, Surviving Sepsis Campaign) and high-quality trials/consensus statements. The ruleset was frozen during 2019–2024; no post-hoc modifications were deployed over the observation window (including after publication of major RCTs), so advice presented to end-users did not change after study start.

**Fig. 2:**
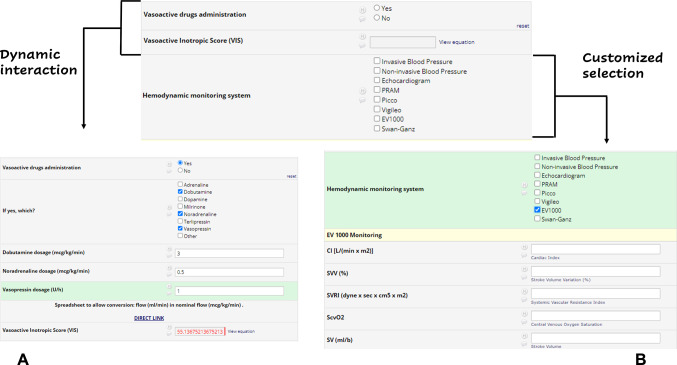
Dynamic decision-support process within the ARRT web platform. Example of dynamic interaction within the web-based platform guiding the management of extracorporeal blood-purification (EBP) therapies. (**A**) When the physician indicates that the patient is receiving vasoactive drugs, the corresponding fields automatically expand for detailed dose entry, and the platform calculates the Vasoactive–Inotropic Score (VIS). (**B**) If hemodynamic monitoring is selected, a dedicated panel appears, allowing the clinician to specify the monitoring system (e.g., PiCCO, Vigileo, EV1000, or Swan–Ganz) and enter related parameters such as cardiac index, stroke volume variation, systemic vascular resistance index, and central venous oxygen saturation. Together, these modules illustrate how the platform provides real-time, adaptive decision support that promotes standardization and accuracy in critical-care practice

As an example, the ARRT Registry suggests readjusting antibiotic posology based on anthropometric patient features, residual organ function, extracorporeal clearance set via EBP, and drug interactions. These multiple and highly complex evaluations require time and multidisciplinary effort to be made at the bedside and to be practically adopted for the patient’s management. The ARRT Registry supports the clinician in the clinical decision-making concerning EBP therapies depending on the experience of the operator, simplifying all these procedural evaluations and allowing the physician to re-adjust continuously all these parameters that might optimize pharmacokinetic/pharmacodynamic (PK/PD) targets for antimicrobials and contribute to eradicate infections and prevent the occurrence of drug resistance. Finally, the registry allows a proactive interaction with the prescribing clinician through personalized algorithms (Fig. 2A and B), guiding several aspects of EBP from the prescription phase until the end of the treatment. From a pragmatic point of view, guidance will: (1) refer to specific guidelines that should be adopted in specific clinical conditions, automatically recognized by the ARRT Registry (these guidelines are provided as references, as pragmatic flow charts for their use, or as a checklist of actions to be followed); (2) suggest interpretations and treatments of complex conditions explored by multiple perspectives, e.g., severe bleeding in patients undergoing EBP with multiple visco-elastic tests (e.g., rotational elastometry) alterations [13].

**Fig. 3:**
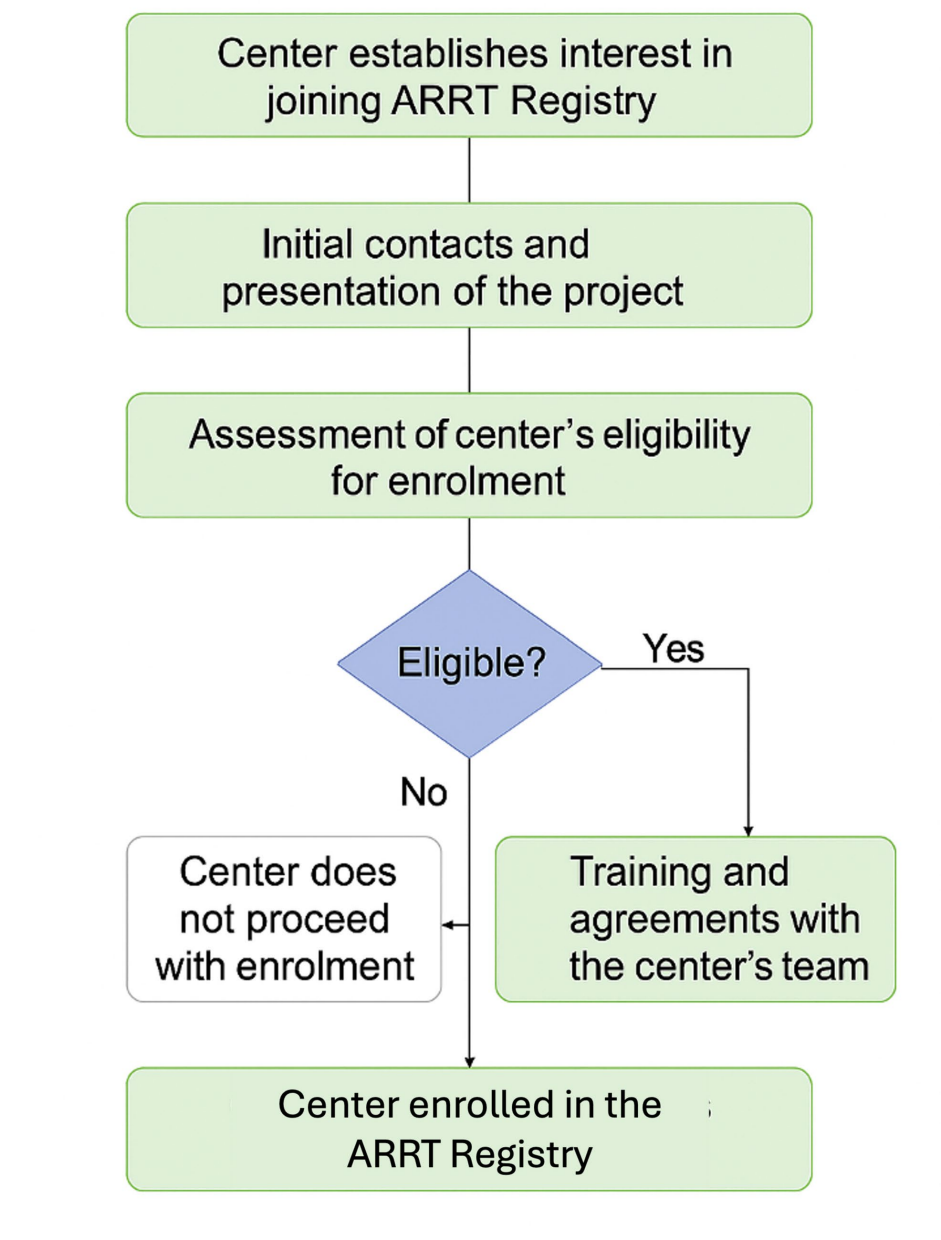
Flowchart of ARRT centre enrolment and quality-improvement cycle. The process for integrating a new centre into the ARRT Registry consists of six main stages: (1) Expression of interest and eligibility confirmation; (2) Appointment of a local principal investigator; (3) Ethical and data-protection approval; (4) Training and credentialing through ARRT Academy modules; (5) Platform activation and test phase; (6) Continuous data submission with feedback reporting and periodic benchmarking reviews. A quality-improvement loop links data entry, feedback, and education to continuous enhancement of clinical practice

From a technical point of view, the ARRT Registry is organized into 13 sections that explore patients’ comorbidities and pathophysiologic data during ICU admission, EBP initiation, prescription, and delivery [14]. It allows monitoring of environmental and short- and long-term patient outcomes, with particular attention to clinical and biochemical trends during the treatment. Its national diffusion has involved 50 cities in Italy in the last three years, counting more than 200 physicians in 68 hospitals: 76% of them are intensivists, while the remaining 24% are nephrologists involved in the care of critically ill patients. To date, 68 public and private centers spread all over Italy are part of the project. The regional distribution (Table 1) includes 18 Centers in Lazio (26.5%), 10 in Toscana (14.7%), 9 in Lombardia (13.2%), 6 in Piemonte (8.8%), 5 in Campania (7.4%), 3 in Puglia (4.4%), 3 in Veneto (4.4%), 2 each in Sicilia, Emilia-Romagna, Calabria, Friuli-Venezia Giulia, Sardegna, and Liguria (2.9% each), and 1 Center respectively in Umbria and Molise (1.5% each) [15]. Program adoption increased over time. See Supplementary Figure S1 for the cumulative number of centers adopting KRT across the observation period. In addition, the platform has been internationalized (GlobalARRT); the main barriers to broader adoption were staff time required for data entry and sustained center engagement. Each new centre follows a standardized enrolment process coordinated by the national ARRT Steering Committee. After expressing interest, the site identifies a local principal investigator responsible for scientific and ethical oversight. The onboarding phase includes verification of ethical and data-protection approval, followed by staff credentialing through a structured training program (ARRT Academy) that combines e-learning modules, webinars, and supervised test sessions on the platform. Once the pilot data-entry phase is successfully completed, the centre is formally activated for routine participation. From that point, data submission is continuous and accompanied by automated feedback reports and periodic benchmarking meetings aimed at sustaining quality improvement and harmonizing clinical practice. This structured enrolment and feedback process, illustrated in Fig. 3, ensures standardization across centres and facilitates both scientific collaboration and professional skill development.

Since its implementation, the ARRT quality-improvement program has led to measurable gains in clinical practice and professional collaboration. A pre-/post-intervention analysis across participating centers showed a significant reduction in missing data (− 20% overall; *p* < 0.001) and improved accuracy of prescription variables, reflecting greater adherence to guideline-based EBP management. In parallel, > 200 professionals from 68 hospitals participated in educational initiatives using the ARRT platform, with > 90% of attendees reporting enhanced competence in CRRT/EBP prescription and monitoring. These preliminary data confirm the registry’s dual role as a clinical-decision and educational tool that standardizes practice and fosters a national learning network.

**Table 1:** Shows the number of centers for each region

Italian Regions with enrolled Centers	Number of enrolled Centers in each Region (percentage)
Calabria	2 (2,9%)
Campania	5 (7,4%)
Emilia-Romagna	2 (2,9%)
Friuli-Venezia Giulia	2 (2,9%)
Lazio	18 (26,5%)
Liguria	2 (2,9%)
Lombardia	9 (13,2%)
Molise	1 (1,5%)
Piemonte	6 (8,8%)
Puglia	3 (4,4%)
Sardegna	2 (2,9%)
Sicilia	2 (2,9%)
Toscana	10 (14,7%)
Umbria	1 (1,5%)
Veneto	3 (4,4%)
*Total*	*68 (100%)*

Between 2019 and 2023, 68 ICUs participated in the ARRT Registry, contributing approximately 686 documented patients treated with extracorporeal blood-purification therapies. Baseline demographic and treatment characteristics of patients included in the ARRT Registry between 2019 and 2023 are summarized in Supplementary Table S1.

According to contemporary epidemiological evidence, sepsis-associated acute kidney injury (SA-AKI) occurs in about 15–16% of ICU admissions, and around 12% of these patients require kidney replacement therapy—corresponding to roughly 2% of all ICU patients receiving extracorporeal therapy [16].

Considering the cumulative ICU capacity of the participating centers (≈ 1 000 beds), the 700 documented patients correspond to an estimated coverage of around 10–15% of all extracorporeal treatments theoretically performed during the study period. This proportion reflects a representative and progressively expanding national adoption of the ARRT Registry across Italian ICUs.

In addition to process indicators, the ARRT Registry captures patient-centred outcomes that reflect the clinical impact of extracorporeal therapies. These include ICU and hospital survival, renal recovery (dialysis independence at discharge), ventilator- and vasopressor-free days, and dynamic scores such as SOFA and VIS. Table S2 summarizes examples of patient-centred outcomes collected and their operational definitions.

Furthermore, the platform provides structured guidance supporting bedside treatment decisions throughout the therapy cycle. As detailed in Table S3, it assists clinicians in selecting the treatment modality, anticoagulation strategy, and initial dose, visualizing circuit performance and hemodynamic trends, and evaluating renal recovery and freedom from kidney-replacement therapy. These features promote standardized, evidence-based decision-making without replacing clinical judgment.

### Combining academic research, National networking, and clinical quality improvement

A clinical/research project has been developed at the University of Florence to reinforce education activities and improve knowledge and expertise on EBP in Italian ICUs engaged in the ARRT Registry.

A three-step methodology has been applied:

1. Each Hospital EBP practice has been evaluated as a baseline.
2. If a lack of knowledge of specific procedures was detected in each Center, specific corrective or preventive actions were implemented.
3. The effect of the actions proposed on patients’ outcomes has been finally evaluated.

This analysis involved EBP users from 14 Centers within Italian ARRT Registry, encompassing a variety of ICU types (68.5% general, 18.5% cardiologic, 7.5% oncology, and 5.5% other) and sizes (54% with 5–10 beds, 34% with 10–20 beds, and the remainder with fewer than 5 beds).

An evaluation of hospital policy and practice regarding EBP and CKRT has been conducted. Practically, we supported each Center in performing a deep analysis of the internal organization (e.g., EBP machines and disposables adopted, skilled team members, or procedures for internal training), stakeholders (industries, universities, or researchers), and expectations guiding different aspects of EBP use (e.g., acquisition, education, retraining, or performances analysis). This analysis has provided an accurate understanding of strengths, weaknesses, opportunities, and threats (SWOT analysis) on the EBP use in each participating Center. While the impact of this program was assessed, detailed findings from this evaluation are part of ongoing work (doctoral thesis) and will be reported in a separate publication.

The lack of consensus in the Literature about clear indications for and effect on outcomes of EBP has a critical role in producing uncertainties regarding EBP use at the bedside [17, 18]. Centers have recognized the expectations for using the ARRT Registry and its role in maintaining control of patient outcomes at the bedside and creating knowledge and evidence in the field of EBP. Interestingly, in most cases, logistic issues related to the stock management policies have limited the variability in the devices (e.g., EBP machines, circuits, or filters) stored by each Center. On the other hand, the lack of internal training has reduced the self-confidence of EBP users in utilizing multiple devices or specific treatments. Both these reasons have affected the Center’s ability to tailor the EBP prescription to the actual needs of each individual patient. In particular, only 45.8% of ICUs have a dedicated warehouse for the stock of EBP devices, and 50% of ICUs have 2 or 3 EBP machines for clinical practice. Centers commonly store and use two different dialytic/replacement solutions; only 20% have four different solutions clinically available at the bedside. Filter options encompass three different disposables in most centers, while in 28.8%, only two different membranes are available.

The SWOT analysis led to the proposal of Center-specific corrective/preventive actions to improve the context in which EBP is used in each Center [19]. Furthermore, results collected among Centers have been analyzed cumulatively to identify topics or issues that recur in different Centers. The lack of educational programs has been identified as the most frequent and severe weakness that limits EBP adoption in the evaluated Centers. Practically, only 15% of Centers undergo yearly training on EBP, while more than 44% do not benefit from any training. Furthermore, nearly 85% of the respondents evaluated the education received at the University as insufficient to obtain the theoretical or practical knowledge for EBP management. This specific issue has led to an extensive update of the academic educational offerings provided in the field of EBP, as outlined below.

## A new era of academic education

The Academic community has promoted continuous education in the field of EBP in recent years. In particular, the University of Florence has implemented and released two different post-graduated academic curricula to provide theoretical knowledge and practical skills for ICU physicians, nurses, and technicians to manage these complex treatments appropriately. The Residency Program in “Anesthesia, Reanimation, Intensive Care and Pain Medicine” (5 years) and the Master’s Degree in “Extracorporeal Blood Purification in Critical care medicine” (1 year) have been implemented with andragogic teaching models aimed at training EBP users with incremental steps of knowledge, from theoretical to technical and practical training.

The learning objective in the first part of these training courses is to strengthen theoretical knowledge on the functioning of machines for blood depuration, the geometric and performance characteristics of different disposables for EBP, and the clinical management of patients undergoing EBP in the intensive care unit.

**Fig. 4:**
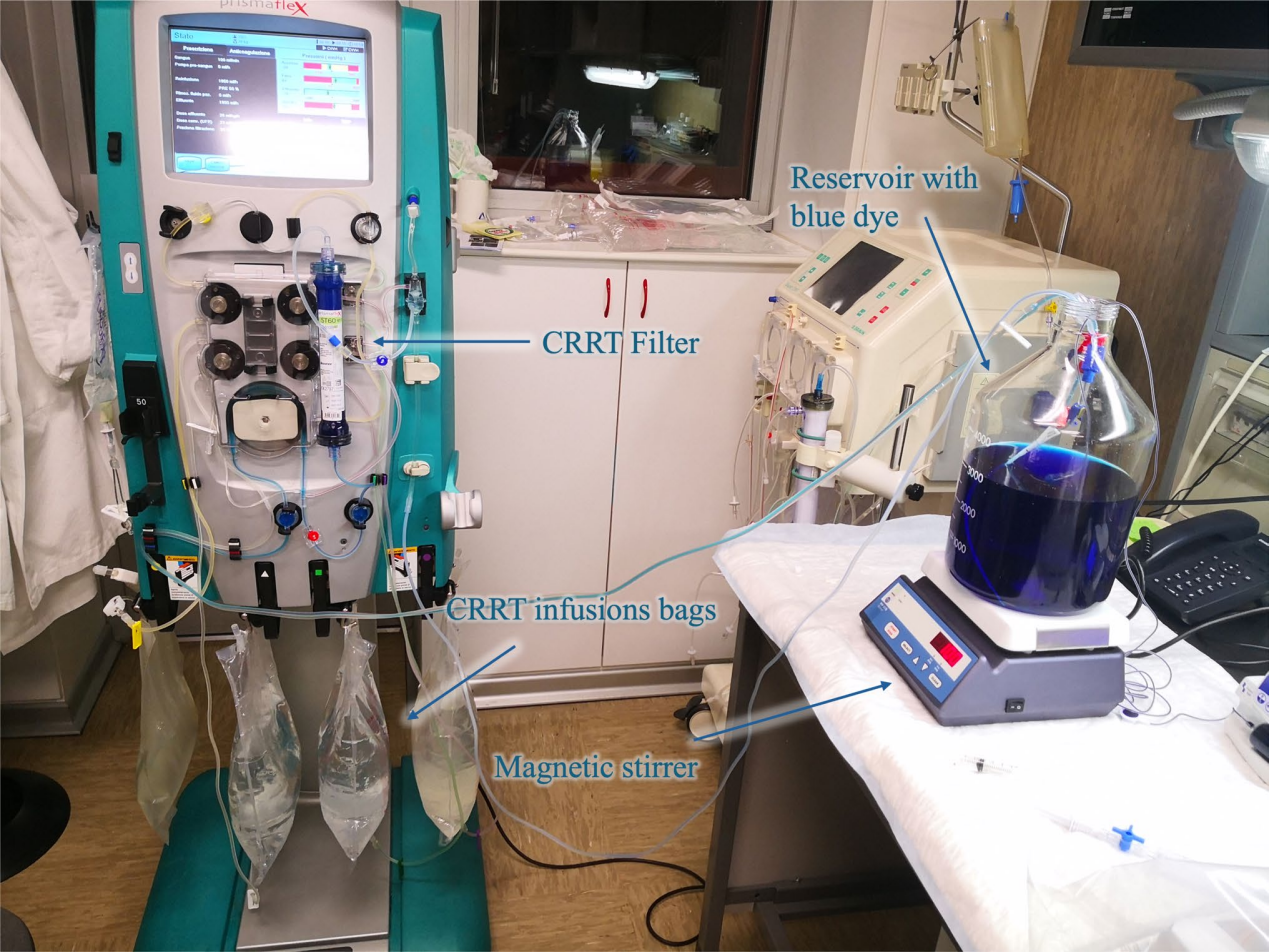
Continuous renal replacement therapy (CRRT) machine and educational setup. Example of a technical and educational setup using a Prismaflex™ system equipped with an ST60 hemofilter. The image illustrates the main components of the extracorporeal circuit, including the CRRT filter, the infusion bags, the reservoir containing blue dye for flow simulation, and the magnetic stirrer used to maintain continuous mixing within the reservoir. This configuration is employed for training and validation purposes to demonstrate extracorporeal circulation, fluid dynamics, and ultrafiltration principles.

In the second part of these training courses, students learn with a practical, exploratory, and controlled “hands-on” approach, how to interact with EBP machines from a technical point of view. It is mainly a systematic exploration of “anatomic features” and “functional properties” of EBP machines and of their methods of use both in conditions similar to those that can be observed during clinical practice and in hyperbolic, extreme conditions (aimed at making evident some practical aspects of the treatment otherwise not appreciated under ordinary conditions of use). In this phase, students understand the technological implications of different treatment phases, strategies, and prescriptions that can be carried out on the machine at the bedside (Fig. 4).

**Fig. 5:**
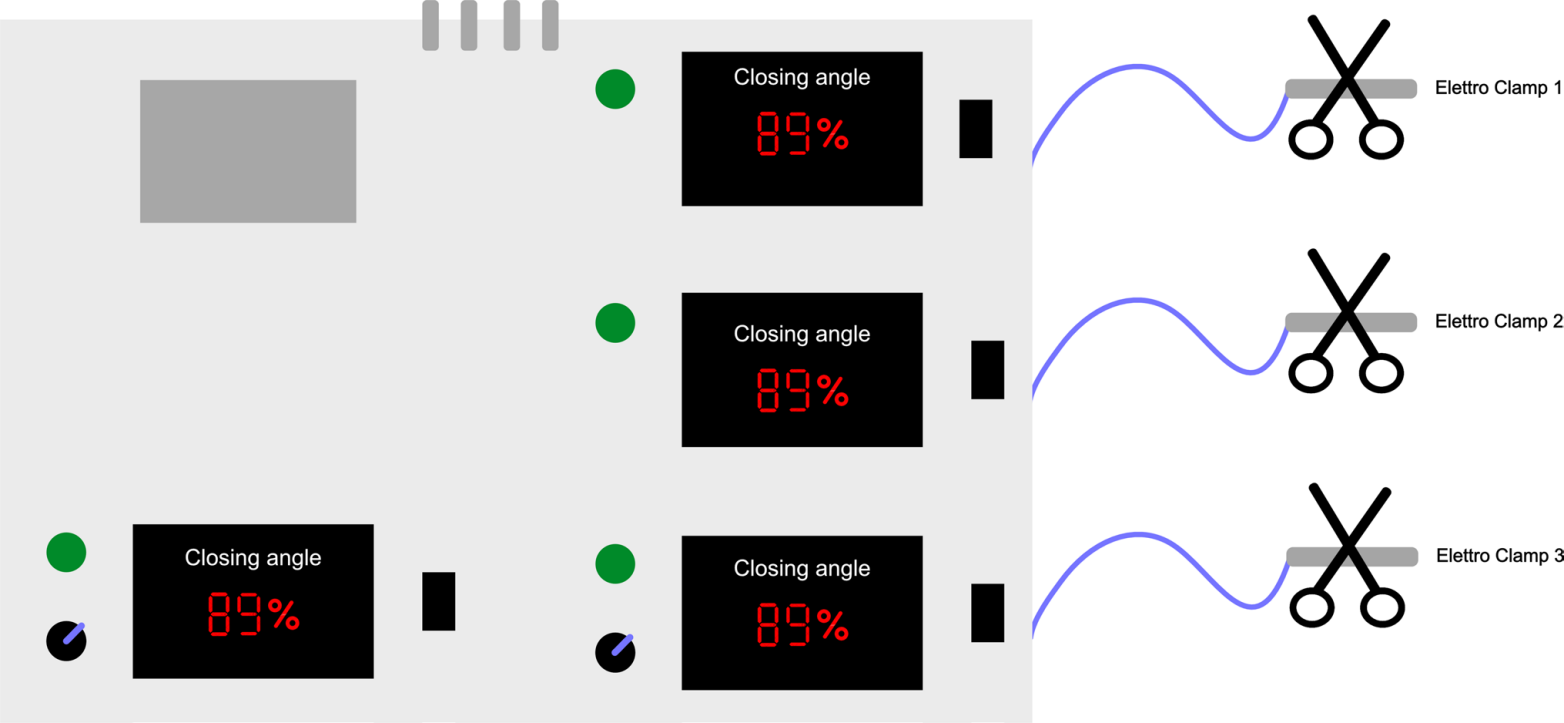
EBP machine influencer. Schematic representation of the electronic clamp control unit (“EBP machine influencer”) used for educational simulation of extracorporeal procedures. The system allows the instructor to modulate the closing angle of each clamp, thereby controlling the degree of line obstruction and triggering specific alarms on the connected EBP machine (e.g., filter clotting, access dysfunction, or pressure imbalance). Each electroclamp is connected to the extracorporeal circuit and remotely operated from the control box, enabling safe, repeatable training scenarios for troubleshooting and alarm interpretation in critical-care settinullngs

Finally, in this second technical phase, the teacher can test the student’s response to the machine’s alarms and troubleshoot by selectively manipulating the lines in the extracorporeal circuit and forcing the EBP machine to produce specific alarms with controlled timing and severity (Fig. 5).

This second technical-oriented teaching phase (i.e., practical despite not being very realistic) is followed by a third one characterized by high-fidelity simulation. The most frequent clinical scenarios and those extremely rare or dangerous for patients are proposed in a safe and controlled immersive high-fidelity experience using a perfect reproduction of an ICU box and a high-tech phantom able to reproduce clinical features observed during critical illness. This technology is also integrated by a system that forces the EBP machine to reproduce specific alarms with remote control, thus preventing alteration of the immersive reality of the simulation.

## Quality improvement, education, and evidence production: a continuum of the same process

The adoption of clinical registries (such as the ARRT Registry), the attention focused on clinical, technical, and environmental data, the objective record of variables and outcomes and a deep understanding of their relationship has a direct effect on the quality of evidence that we can provide nowadays in the field of EBP.

Several limitations hinder the ability of randomized controlled trials (RCTs) to provide reliable results in the critical care setting. RCTs are often designed with homogeneous patient populations and exclude patients with multiple comorbidities [20]; this may limit biases due to multiple pathophysiologic derangements of critical illness. Strict inclusion and exclusion criteria limit the applicability of clinical findings to patients with complex medical histories and their generalizability to a broader population of critical care patients. Ethical concerns preclude the enrollment of incompetent patients in RCTs. Unfortunately, most of the patients undergoing EBP are in these conditions. Finally, large and expensive RCTs have failed due to underpowering or wrong endpoints [21].

For all these reasons, evidence on the EBP use came from observational cohort studies with limited patients. As new and more robust evidence in this field is needed, real-world evidence from patient registries is increasingly being adopted to complement the findings of clinical trials [22]. These new tools have several advantages over clinical trials in critical care. Real-world trials can provide a complete picture of a therapy’s applications and outcomes by including a more diverse patient population with a broader range of comorbidities and disease severities [23]. Furthermore, registries can follow patients over extended periods, providing insights into the long-term impact of therapy on outcomes of interest. National and international-wide clinical registries are becoming more prevalent, providing valuable information and enhancing clinical guidance for diseases where prospective randomized trials are missing or difficult to conduct [24]. Registry-based trials allow to identify sub-populations of patients who may benefit most from therapy within a restrained budget [25]. It must be acknowledged, however, that causal associations cannot be inferred with this methodology, even if recent statistical attempts are being based on big observational data (i.e., Directed Acyclic Graph). So far, RCT studies remain the only way to verify causal associations between treatments and outcomes.

Furthermore, it is important to highlight that knowledge and practice surrounding EBP in KRT likely vary globally due to differing models of professional responsibility between intensivists and non-intensivists [26], and even within countries like Italy, regional variations in KRT prescription responsibilities exist. Registry usage metrics and clinician adherence/benefit analyses were collected as part of the quality‑improvement program and will be reported separately.

Data from 623 critically ill patients have been collected in the ARRT Registry [15]. The status of each patient is monitored for 10 days, from ICU admission to discharge. Among Centers involved in the ARRT Registry, 23 are also using this ICT tool for research and publication purposes with the approval of their own Institutional Review Board (IRB): Toscana (6), Lombardia (4), Lazio (4), Campania (2), Veneto (2), Piemonte (2), Puglia (1), Umbria (1), Calabria (1). The ARRT Registry, defining possible clusters of critically ill patients treated with EBP therapies, can help set targeted EBPs, known as “precision medicine,” for specific patients who benefit most from a specific extracorporeal therapy [14]. This approach is a helpful way to design successful RCTs that show the efficacy of a treatment on specific sub-populations of critically ill patients. In the recent pandemic, the ARRT Registry collected a considerable amount of data, leading to the publication of preliminary evidence on the feasibility and safety of using EBPs in patients affected by severe COVID-19 [25]. We can expect more evidence from this large National Registry, with the possibility of designing and planning future RCTs on the efficacy of EBPs [8].

## Conclusion

EBP treatments are complex. Several reasons lead to uncertainties regarding their use, such as the unavailability of instruments that objectively analyze real-world practice, inadequate training for adopters, and incomplete evidence in the Literature. A safety and quality improvement program focused on EBP has been implemented in Italy by a national network of EBP users involved in a multicenter national program called ARRT Registry. A deep analysis of reasons leading to inadequate management or uncertainties at the bedside has been conducted, and the lack of technical and non-technical skills has been recognized as a primary factor. The provision of training and education in the field of EBPs has been profoundly reformed to provide users with the necessary skills to offer patients the most appropriate treatments at the bedside. Finally, from the intersection of quality programs, education activities, and objective outcome measurement, a new model has evolved through which research in the field of EBPs can ultimately add on patient registries, real-life data, and deep learning analyses to expensive and seldom conclusive RCTs.

## Supplementary Information

Below is the link to the electronic supplementary material.

Supplementary Material 1

## Data Availability

Data are securely stored in the database of the Registry (www.arrt.eu). Data are provided upon request to the PI.
