# Supplementary material for: Program of quality improvement for extracorporeal blood purification therapies in the intensive care unit
Source: J Clin Monit Comput. 2025 Dec 12;40(3):735–45. doi: 10.1007/s10877-025-01396-7 (PMC13194202; doi:10.1007/s10877-025-01396-7)
Supplement: Supplementary file 1 — Supplementary Material 1 [file 10877_2025_1396_MOESM1_ESM.docx]

**Supplementary Materials**

**Title:** Program of quality improvement for extracorporeal blood purification therapies in the intensive care unit

**Authors:** Matteo Cecchi^1^, Diego Pomaré Montin^1^, Antonio Fioccola^1,2^, Vittorio Bocciero^1^, Caterina Scirè Calabrisotto^1^, Filomena Autieri^3^, Manuela Benelli^3^, Andrea Geppetti^4^, Zaccaria Ricci^1,5^, Stefano Romagnoli^1,6^ and Gianluca Villa^1,6^

**Authors’ affiliation:**

^1^ Department of Health Sciences, Section of Anesthesiology, Intensive Care and Pain Medicine, University of Florence, Florence, Italy

^2^ Department of Anesthesia and Intensive Care, ASST Santi Paolo e Carlo, San Paolo University Hospital Milan, Italy

^3^ Unit of Accreditation, Quality and Risk Management, AOU Careggi, Florence, Italy

^4^ Department of Civil and Environmental Engineering, University of Florence, Florence, Italy

^5^ Department of Anesthesia and Critical Care, Meyer Children’s University Hospital, IRCCS, Florence, Italy

^6^ Department of Anesthesia and Intensive Care, Section of Oncological Anesthesia and Intensive Care, AOU Careggi, Florence, Italy

**Corresponding author:**

Gianluca Villa

Department of Health Sciences, Section of Anesthesiology, Intensive Care and Pain Medicine, University of Florence, Florence, Italy and Department of Anesthesia and Intensive Care, Section of Oncological Anesthesia and Intensive Care, AOU Careggi, Florence, Italy

e-mail: [gianluca.villa@unifi.it](mailto:gianluca.villa@unifi.it)

Summary of Supplementary Figures and Tables

[Figure S1. Cumulative number of centers adopting ARRT (Nov 2020–Sep 2023) 3](#_Toc213946146)

[Table S1. Baseline characteristics of patients and extracorporeal treatments documented in the ARRT Registry 4](#_Toc213946147)

[Table S2. Examples of patient-centred outcomes collected in the ARRT Registry 5](#_Toc213946148)

[Table S3. Examples of treatment decisions supported by the ARRT Registry 6](#_Toc213946149)

## ****Figure S1. Cumulative number of centers adopting ARRT (Nov 2020–Sep 2023)****


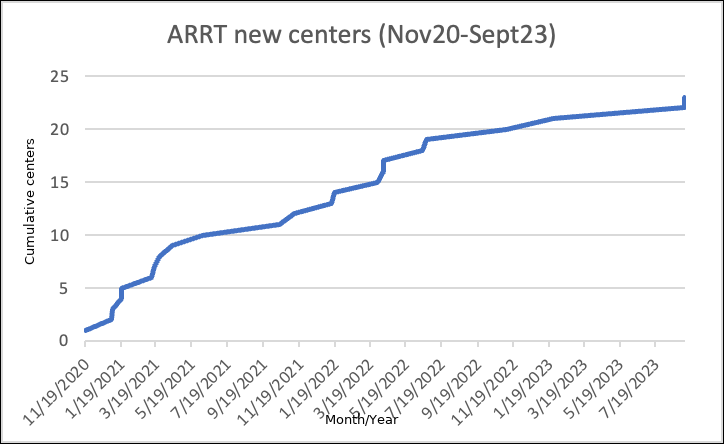


*Figure S1. Cumulative number of centers adopting ARRT (Nov 2020–Sep 2023). Line plot showing the cumulative count of participating centers over time. Data source: ARRT Registry onboarding logs. Note: This figure reflects cumulative adoption and does not imply patient-level outcomes.*

## ****Table S1. Baseline characteristics of patients and extracorporeal treatments documented in the ARRT Registry****

| **Category \|** Variable | N (%) or mean ± SD | **Category \|** Variable | N (%) or mean ± SD |
| --- | --- | --- | --- |
|  |  |  |  |
| **Patients** |  | **Treatments** |  |
| N (%) | 686 (100) | **Treatment target** |  |
| Sex (Female), N (%) | 187 (27.2) | *Renal substitution, N (%)* | 312 (45.5) |
| Sex (Male), N (%) | 499 (72.8) | *Immunomodulation, N (%)* | 76 (11.1) |
| BMI (kg/m^2^) | 28.2 ± 6.3 | *Both, N (%)* | 298 (43.4) |
| **Admission reason** |  | **Hemodiafilter** |  |
| *Medical, N (%)* | 461 (67.2) | *AN69ST, N (%)* | 168 (24.5) |
| *Post-Surgical, N (%)* | 218 (31.8) | *oXiris, N (%)* | 289 (42.1) |
| *NA, N (%)* | 7 (1.0) | *Septex, N (%)* | 39 (5.7) |
| **CKD stage** |  | *Others, N (%)* | 190 (27.7) |
| *1, N (%)* | 37 (5.4) | **Anticoagulation** |  |
| *2, N (%)* | 61 (8.9) | *RCA, N (%)* | 356 (51.9) |
| *3, N (%)* | 124 (18.1) | *Systemic heparin, N (%)* | 216 (31.5) |
| *4, N (%)* | 89 (13.0) | *Regional heparin-protamine, N (%)* | 5 (0.7) |
| *5, N (%)* | 101 (14.7) | *None, N (%)* | 60 (8.7) |
| *NA, N (%)* | 274 (39.9) | *NA, N (%)* | 49 (7.2) |

*Table S1 summarizes the baseline demographic, clinical, and treatment characteristics of patients enrolled in the ARRT Registry between 2019 and 2023. These data provide a descriptive overview of the population receiving extracorporeal blood-purification therapies, including variables such as sex, body-mass index, admission category, chronic kidney disease stage, treatment target, hemofilter type, and anticoagulation strategy. This standardized dataset defines the registry’s patient cohort and serves as the reference population for subsequent analyses of outcomes and treatment performance. N, Number; BMI, body mass index; kg, kilograms; m², meter-square; RCA, regional citrate anticoagulation. Data refer to the 686 patients included in the ARRT Registry (2019–2023). Variables include demographic, clinical, and treatment characteristics recorded at the start of extracorporeal therapy. Continuous variables are expressed as mean ± standard deviation, and categorical variables as n (%).*

## ****Table S2. Examples of patient-centred outcomes collected in the ARRT Registry****

|  | **Outcome examples** | **Operational definition / data source** |
| --- | --- | --- |
| **Survival and recovery** | • ICU survival  • Hospital survival | Binary outcomes derived from ICU and hospital discharge records within the ARRT platform |
| **Renal recovery** | • Dialysis independence at ICU or hospital discharge | Cessation of kidney replacement therapy with spontaneous urine output >400 mL/day and serum creatinine <2 mg/dL |
| **Organ-support dependency** | • Ventilator-free days  • Vasopressor-free days | Calculated from daily treatment records (mechanical ventilation and vasoactive support modules) |
| **Physiological stabilization** | • ΔSOFA score over treatment course  • VIS reduction ≥ 20 % from baseline | Derived from automatically calculated scoring systems embedded in ARRT |
| **Treatment burden and safety** | • Circuit lifespan  • Major adverse events (bleeding, hypothermia, access dysfunction) | Recorded as structured variables for each extracorporeal session |

Table S2 shows examples of patient-centred outcomes routinely collected within the ARRT Registry. These indicators complement process and technical variables by capturing the clinical impact of extracorporeal blood-purification therapies. Outcomes are automatically derived from structured case-report forms or validated calculation modules (SOFA, VIS) and provide a standardized framework for benchmarking clinical performance across participating ICUs.

## ****Table S3. Examples of treatment decisions supported by the ARRT Registry****

| **Phase of care** | **Examples of decisions supported** | **Mechanism within ARRT platform** |
| --- | --- | --- |
| **Prescription phase** | • Selection of treatment modality (e.g., continuous vs adsorption)  • Choice of anticoagulation (e.g., citrate vs heparin)  • Initial effluent-dose setting (mL/kg/h) | Automated calculators and checklists based on KDIGO criteria, hemodynamic profile, and patient characteristics |
| **Monitoring and adjustment phase** | • Filter change or circuit optimization  • Reassessment of hemodynamic stability (VIS trend)  • Review of antimicrobial dosing in relation to extracorporeal clearance | Real-time dashboards visualize circuit performance and recalculated scores, prompting review of efficiency or pharmacologic adequacy |
| **Evaluation phase** | • Assessment of renal recovery and freedom from kidney-replacement therapy  • Evaluation of organ-support trends (e.g., SOFA, fluid balance) | Longitudinal data visualization supports clinical judgment regarding treatment continuation or discontinuation |

Table S3 summarizes examples of clinical decisions supported by the ARRT Registry at different phases of the extracorporeal-therapy cycle. The platform provides interactive tools for prescription, monitoring, and evaluation, encouraging timely reassessment of therapy settings and standardized, evidence-based decision-making while preserving clinician autonomy.
